# Learning curve and patient outcomes in gallstone surgery: a national register-based cohort study

**DOI:** 10.1007/s00464-026-13073-0

**Published:** 2026-07-20

**Authors:** Nathalie Young, Lars Enochsson, Hans Pettersson, Peter Elbe, Ninos Oussi, Gabriel Sandblom

**Affiliations:** 1 Department of Clinical Science and Education, Karolinska Institutet Södersjukhuset, Stockholm, Sweden; 2 S1 KI SS Research and Education, Södersjukhuset, 118 83 Stockholm, Sweden; 3 Department of Surgery, Södersjukhuset, Stockholm, Sweden; 4 Department of Clinical Science, Intervention and Technology, Karolinska Institutet, Stockholm, Sweden; 5 Department of Clinical Science and Education, Södersjukhuset Karolinska Institutet, Stockholm, Sweden; 6 Department of Digestive Diseases, Karolinska University Hospital, Stockholm, Sweden; 7 Centre for Clinical Research, Region Sörmland, Uppsala University, Eskilstuna, Sweden; 8 Department of Clinical Science, Intervention and Technology, Division of Urology, Karolinska Institutet, Stockholm, Sweden

**Keywords:** Laparoscopic cholecystectomy, Learning curve, Extended learning curve, Operative time, Complications, Registry study

## Abstract

**Background:**

There is no consensus on, or a defined minimum number of surgical laparoscopic cholecystectomies required to be considered an independent surgeon. The aim of the present study was to assess how surgical outcomes improve with surgical volume.

**Method:**

Data on laparoscopic cholecystectomies performed by surgical trainees and surgeons between 2006 and 2022 were retrieved from the Swedish national register for gallstone surgery (GallRiks). Surgeons who had not registered the number of procedures performed before first GallRiks entry were excluded. Comparisons regarding operation time and postoperatively observed complications were divided into strata of 50 performed procedures.

**Results:**

Between 2006 and 2022, 139,010 cholecystectomies were performed by surgeons (*n* = 1664) who had not yet performed 2000 procedures. The mean operation time decreased from 111 min (95% confidence interval (CI) 111–112 min) to 75 min (95% CI 73–77 min) after 800 completed procedures. During the first 50 procedures, the incidence of postoperatively observed complications was 4.7 and 7.0%, respectively, if the cholecystectomy was performed with or without a registered assistant (*p* = 0.007). The difference in the incidence of complications for assisted and unassisted procedures remained statistically significant up to 250 interventions performed.

**Conclusion:**

Improvement in surgical outcomes and shorter operation time were observed over a long course of the expected learning curve. Although the presence of an assistant had a significant impact on the outcome during the initial phase of the learning curve, we do not know to what extent the assistant took part in the procedure. In GallRiks, only the presence of an assistant is registered, not what the assistant did during the procedure.

**Graphical abstract:**

**Figure:**
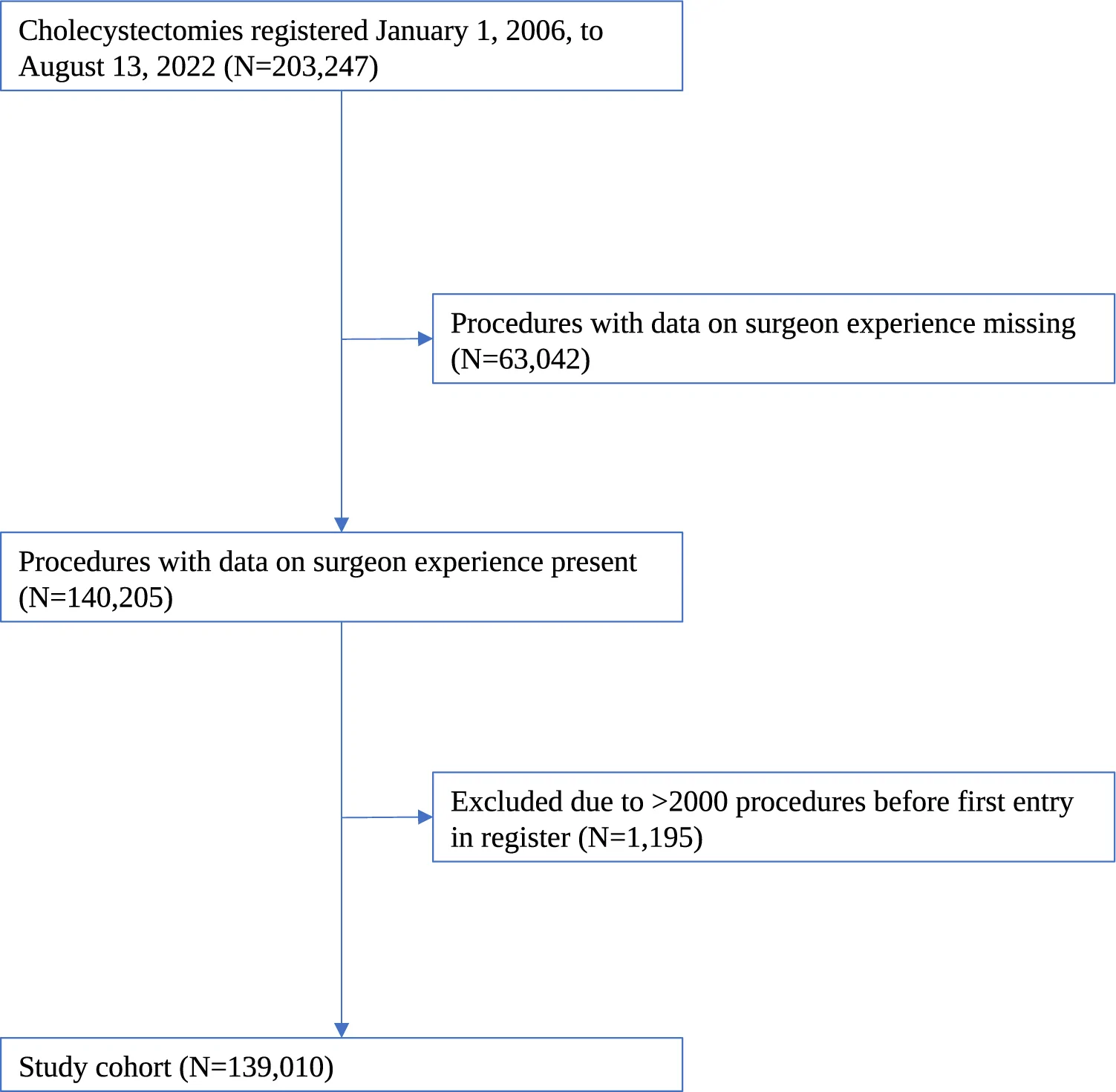

**Supplementary Information:**

The online version contains supplementary material available at https://doi.org/10.1007/s00464-026-13073-0.

Laparoscopic cholecystectomy, established as the gold standard for gallbladder surgery since the late 1980s, offers reduced hospital stays and lower complication rates, such as surgical site infections and incisional hernias, compared to open surgery [1, 2]. Despite these benefits, the procedure demands significant technical skills, and awareness of the risks of complications, such as bile duct injuries or bleeding [3]. Thus, proper training and understanding the learning curve are critical for ensuring patient safety and optimising outcomes [4]. The learning curve for general surgery trainees involves repeated practice, starting with supervised tasks and progressing to independent surgery. This curve typically features a steep initial phase of rapid improvement, followed by a slower rate of progress, eventually plateauing where further improvements are minimal [5–8].

No universally accepted standard exists for the minimum number of laparoscopic cholecystectomies required to achieve independent surgeon status or an acceptable complication rate during the learning phase. The Swedish Surgical Society recommends a minimum of 20 elective procedures for trainees, based on expert consensus [9]. Some studies suggest 20–30 procedures are needed for operation time and complication rates to stabilise, whilst others, like Williams et al. (2019), indicate 20 observations are required just to achieve a reproducible performance score amongst surgical trainees [10]. U.S. guidelines recommend at least 50 procedures during residency to ensure competence for routine and moderately complex cholecystectomy cases [11]. Higher surgical volumes are generally linked to better outcomes [12], with Swedish registry data reporting bile duct injury rates of 0.4% for elective and 0.5% for acute cholecystectomies [13–15]. Surgeons performing fewer than 10–20 procedures annually have higher complication rates compared to those performing 50–100 [16]. This study aims to explore the correlation between surgical volume and patient outcomes in a Swedish context.

## Methods

The study was based on data from GallRiks (the Swedish registry for gallbladder surgery and endoscopic retrograde cholangiopancreatography (ERCP)). Ethical approval number 2022-04919-01. The registry was established in May 2005 and covers more than 90% of all cholecystectomies carried out in Sweden [17]. Variables registered in GallRiks include, amongst others, sex, age, operation indication, surgical technique, American Society of Anaesthesiologists (ASA) classification and body mass index (BMI). The timeline of each individual surgeon is traceable, even if transferring between different units in Sweden. Prior to a surgeon’s first entry, the number of procedures must be stated.

This was a retrospective observational cohort study based on prospectively collected data from GallRiks. Healthcare data were collected from the GallRiks register between 2006 and 2022 to investigate the relationship between surgical volume, operation time and patient outcomes for surgical trainees and surgeons. Abscesses included surgical site infections in the subcutaneous tissue as well as deep abscesses caused by contamination of the operative field. Bile duct injury was considered any tissue damage to the wall of any bile duct in the biliary tree detected during the procedure or postoperative period. The criterion for bleeding was any bleeding requiring blood transfusion or active intervention to stop the bleeding. Procedures were stratified by increments of 50 performed cholecystectomies to assess proficiency and outcomes. The number of procedures previously performed by the responsible surgeon was considered as exposure. This included procedures prior to the first GallRiks entry as well as all procedures included in the register. The formal position of the surgeon was not included in the analysis; only accumulated number of procedures was considered.

A multivariable logistic regression analysis was performed to study the association of the total accumulated procedure volume, the number of procedures performed in the year before each procedure, and the presence of an assistant as covariates. A multivariable linear regression analysis was performed with mean operation time as outcome and the same variables as covariates. The study included all surgeons and trainees who reported their prior procedure count at their first GallRiks registration. Exclusions applied to surgeons with 2000 or more performed cholecystectomies before data collection and those with unreported prior procedures. Both elective and acute cholecystectomies were included. The primary outcome was operation time (from first incision to last stitch, including intraoperative ERCP if performed). The secondary outcome was surgical complications. Independent factors included ASA physical status, supervised procedure status, acute cholecystitis, patient age, and procedure type (elective or acute). Procedures with multiple surgeons, including a specialist in surgery were classified as supervised. The assistant could be a trainee or a surgeon. Operation time was compared using a two-sample t-test, and surgical complications were analysed with a chi-square test. Classification trees, built with the chi-square automatic interaction detector (CHAID) algorithm, identified factors associated with surgical complications (abscess, bleeding or bile leakage) and operation time, grouping patients into distinct nodes based on similarity [18, 19].

## Results

**Fig. 1:**
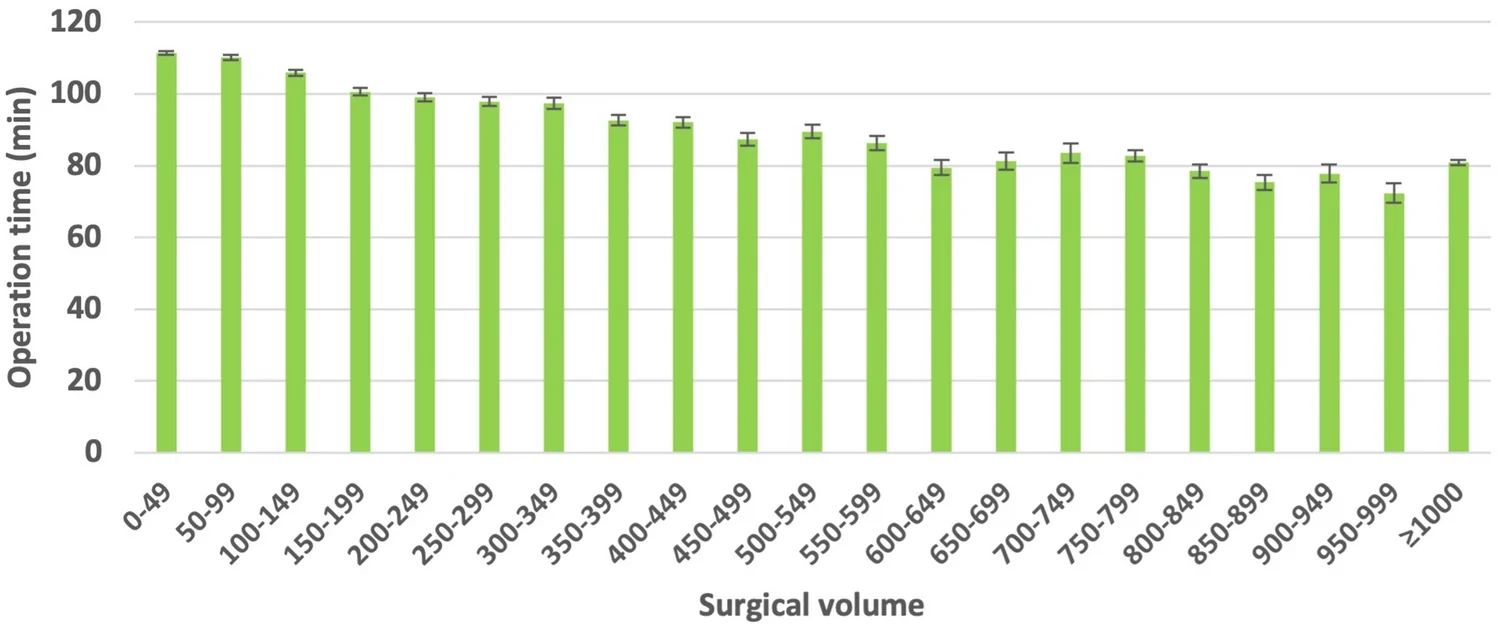
Mean operation time for gallstone surgery (cholecystectomy) decreases as a surgeon’s experience increases, measured by cumulative surgical volume (error bars: 95% CI)

**Fig. 2:**
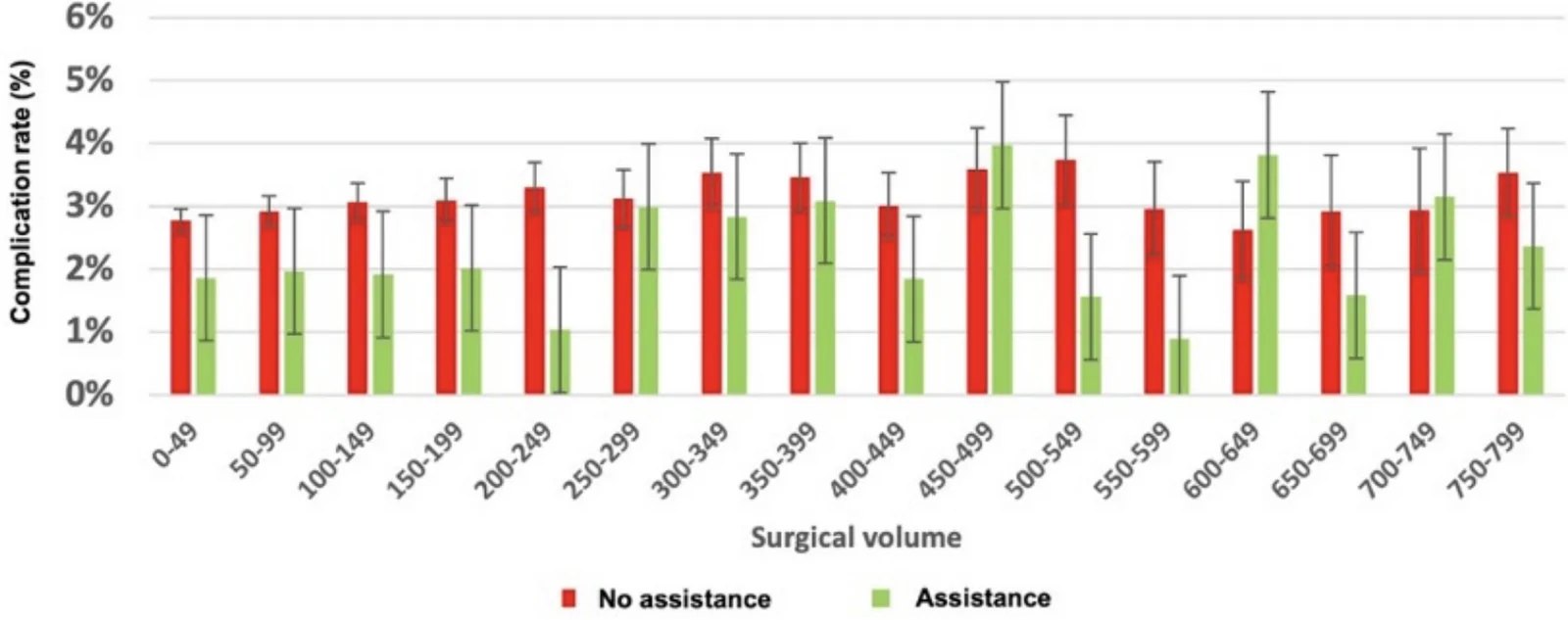
Patient outcome (complication rate) by surgical volume and with or without assistance from another surgeon (error bars: 95% CI)

Between 2006 and 2022, 139,010 cholecystectomies were performed by 1664 surgeons who had not yet performed 2000 procedures. Patient characteristics are shown in Table 1. Evaluating the factors influencing operation time, the CHAID tree diagram identified surgical approach, acute cholecystectomy, surgical volume and the presence of an assistant as primary predictors. The first split was the surgical approach (laparoscopic cholecystectomy was faster with a mean operation time of 93 min). The second split showed that acute cholecystitis cases took longer time compared to elective cases. The third split showed that mean operation time decreased with increased surgical volume for both acute and elective cholecystectomies. (Online Appendix 1). Two histograms further show the relationship between surgical volume, operation time, and the use of assistants across varying levels of surgeon experience. The CHAID tree diagram showed that the laparoscopic approach was faster, as was an elective procedure with an assistant. Altogether, *n* = 6004 (4.3%) of the procedures were performed with an assistant present. The mean operation time decreased from 111 min (95% CI 111–112) to 75 min (95% CI 73–77) after 800 cholecystectomies (Fig. 1). Operation time decreased with increasing experience. This decline reflects a steeper initial learning phase (0–200 procedures, dropping from 100 to 85 min), followed by a slower improvement phase (200–800 procedures from 85 to 75 min). Beyond 800 procedures, operation time stabilised at 70–80 min indicating a plateau phase where further experience yielded minimal improvements. Variability in operation time as indicated by error bars was higher at lower surgical volumes and decreased with experience, suggesting greater consistency amongst experienced surgeons. During the first 50 procedures, the incidence of postoperatively observed complications was 4.7 and 7.0% respectively, if the cholecystectomy was performed with or without a registered assistant (*p* = 0.007). The difference in the complication rate for assisted and unassisted procedures remained statistically significant up to 250 procedures. The proportion of surgeries performed with or without an assistant across surgical volumes was also examined (Fig. 2). In a multivariable logistic regression analysis, the total accumulated volume of procedures (Odds Ratio [OR] 0.993, 95% confidence interval [CI] 0.991–0.994), the number of procedures performed the year before each procedure (OR 1.011, CI 1.009–1.013), presence of assisting non-specialist (OR 0.557, CI 0.427–0.727) and presence of assisting specialist (OR 0.719, CI 0.605–0.855) were significantly associated with the risk of postoperative complication (all *p* < 0.001). Similarly, in a multivariable linear regression analysis, the same covariates were significantly associated with operation time (all *p* < 0.001).

**Table 1:**
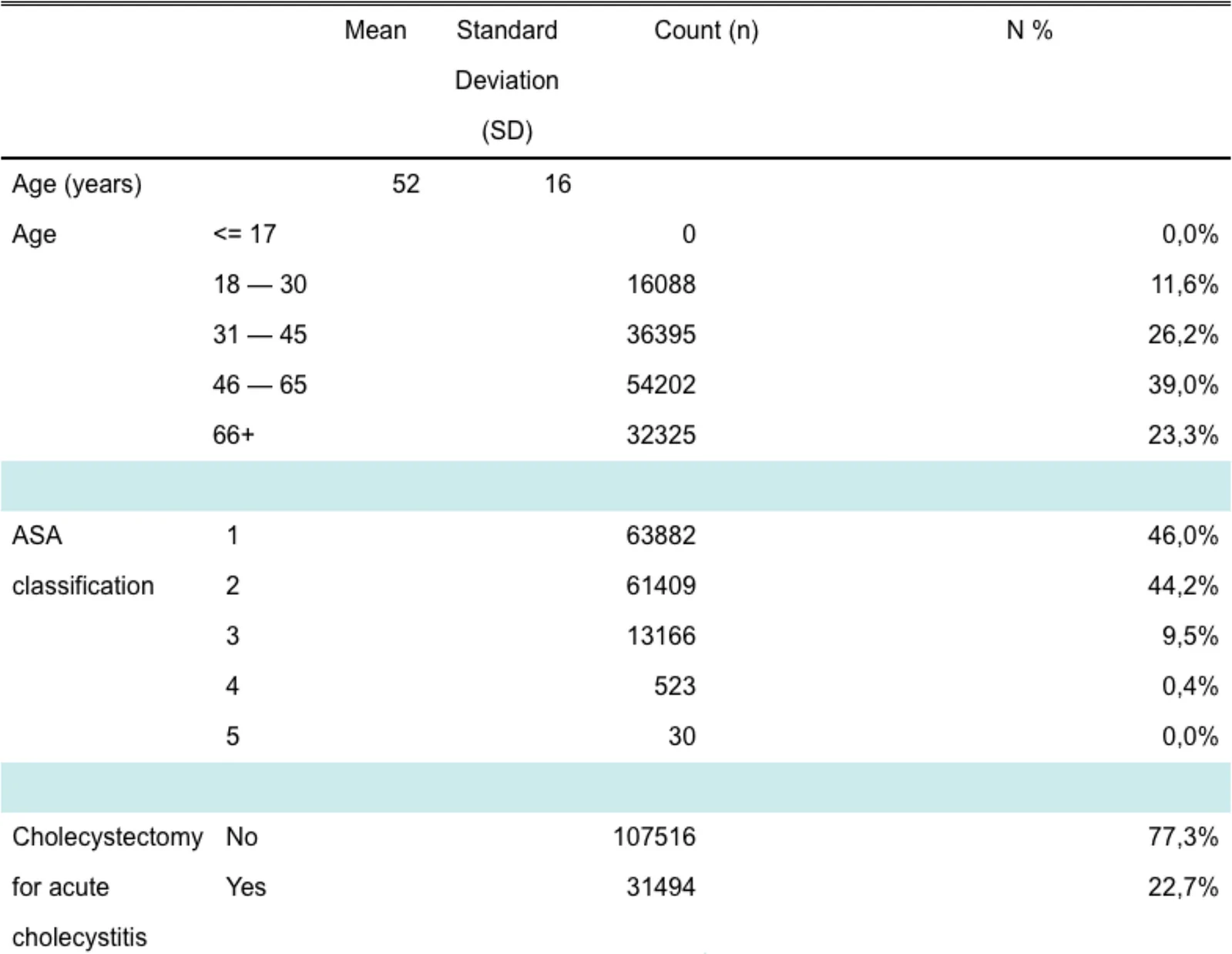
Patient characteristics

In subgroup analysis of procedures performed for acute cholecystitis without stratification for presence of an assistant, it was found that the complication rate decreased over time and that approximately 600 procedures were required to reach a complication rate not higher than 3.0%.

At lower surgical volumes (0–199 procedures), most surgeries were performed without an assistant with a complication rate ranging from 2.5 to 3.5% compared to 1 to 2% with an assistant. Assistant presence peaked at 350–399 procedures, reaching approximately 3%, nearly matching the proportion without an assistant around 3.5%. This peak corresponds to the slower improvement phase in operation time (200–500 procedures), suggesting that assistants may facilitate efficiency gains during this transitional period. Beyond 500 procedures, presence of assistant was around 1.5 to 2.5%, whilst the proportions without an assistant remained slightly higher at 2 to 3%, indicating that experienced surgeons may operate more independently once they reach peak efficiency.

## Discussion

This population-based cohort study demonstrates that the learning curve for surgeons performing gallstone surgery can be quantified by operation time and complication rate. The results demonstrate a significant reduction in operation time, dropping from an average of 111 min to 75 min after 800 completed laparoscopic cholecystectomies. This indicates a prolonged learning curve, extending well beyond the initial training phase and into years of independent practice. Proficiency, as measured by operation time, stabilises only after a high volume of procedures. This contrasts with prior studies suggesting sufficient proficiency after 20–50 cases [20]. This discrepancy may reflect differences in how proficiency is defined—earlier studies often focussed on basic competence or complication rates, whereas this study emphasised efficiency (operation time) as a marker of mastery. The extended learning curve implies that surgical trainees and early—career surgeons may require more extensive experience than current Swedish surgical trainee guidelines (minimum of 20 procedures) anticipate reaching peak efficiency. The guideline recommendation of a minimum of 20 procedures excludes acute cholecystitis. As expected, the study highlights that operative time was longer in cases involving open cholecystectomy, acute cholecystitis, or the absence of an assistant. These findings align with clinical expectations, as open procedures and acute cases are inherently more complex, and the lack of assistance may slow workflow or increase the cognitive load on the primary surgeon [17]. The persistence of longer operation times in unassisted procedures up to 250 procedures suggests that the presence of a second surgeon or assistant plays a critical role in streamlining the procedure during the early-to-mid stages of a surgeon’s career. Not only the totally accumulated volume had an impact on the outcome, but also the intensity of training. A high number of procedures performed prior to the procedure significantly predicted the outcome, suggesting that concentrating training within a limited period has a greater impact on progress than spreading it over a longer period. The incidence of postoperative complications was notably higher during the first 50 procedures (4.7% with assistance vs. 7.0% without, *p* = 0.007), with a statistically significant difference persisting up to 250 procedures. The multivariable logistic regression analysis showed that the experience of the assistant influenced the outcome. The risk of complications was, however, greater in case an experienced colleague assisted. This is probably due to a selection bias, as more experienced surgeons may have been called for in case a complicated situation occurred. This underscores the importance of having an assistant/second surgeon during the early phases of a surgeon’s development. Laparoscopic cholecystectomy for acute cholecystitis is considered a procedure with longer learning curve than for planned laparoscopic cholecystectomy in case of uncomplicated gallstone disease. In the present cohort, it was found that 600 procedures were required to reach an acceptable complication rate in case of acute cholecystitis. Studies with more granulated data are needed to confirm this. The higher complication rate in unassisted procedures aligns with previous research linking lower surgical volume and lack of experience to adverse outcomes, such as bile duct injuries. Whilst the complication rate decline was less dramatic than the reduction in operative time, the sustained benefit of assistance up to 250 procedures suggests that independent practice should be deferred until a surgeon has substantial supervised experience. Given these findings, the Swedish Surgical Society’s recommendation of a minimum of 20 elective laparoscopic cholecystectomies for trainees appears insufficient. The U.S guideline of 50 procedures aligns more closely with the point at which complication rates begin to stabilise, though even this may not suffice for achieving efficiency, as operation time plateaus at 800 procedures. High-volume surgeons with about 50–100 procedures annually consistently show better outcomes in prior studies, and this study supports that trend, with complication rates and operative times improving with greater experience. However, the plateau at 800 procedures suggests that true mastery may require a decade or more of practice, especially in lower volume settings, which include most surgical departments in Sweden performing cholecystectomies. The considered high-volume centres in Sweden estimate around 50–80 cases per surgeon annually. Data from this study suggest that surgical trainees or surgeons with fewer than 50 laparoscopic cholecystectomy procedures should not perform these operations without an assistant. This raises questions regarding the safe minimum number of procedures annually per surgeon without affecting patient outcomes. These findings have significant implications for surgical education and healthcare policy. First, the prolonged learning curve suggests that training programmes should prioritise access to higher surgical volumes, potentially through longer uninterrupted rotation at a high-volume centre when learning cholecystectomies, starting with elective cases. This could include facilitating mandatory training time in simulation-based environments. The protective effect of having an assistant up to 250 procedures calls for structured mentorship programmes for early-career surgeons, ensuring they are not thrust into independent practice prematurely. Moreover, the correlation between volume and outcomes supports the designation of high-volume centres for complex cases, though this presents a challenge in balancing a broader general surgery concept against sub specialisation.

The study’s strength is its substantial sample size (139,010 procedures) and extensive observation period (2006–2022), both derived from a comprehensive national registry. This enhances the reliability and generalisability of the findings within Sweden. The use of classification trees to identify factors like acute cholecystitis and supervision adds nuance to the analysis. However, limitations include the retrospective design, which may miss unrecorded confounders. Potential sources of residual confounding are surgeon fatigue or patient comorbidities not captured in ASA scores.

Although the presence of an assistant had a significant impact on the outcome during the initial phase of the learning curve, we do not know to what extent the assistant took part in the procedure. In GallRiks, only the presence of an assistant is registered, not what the assistant did during the procedure. The criteria for complications did not unequivocally reflect unfavourable outcomes directly related to the surgical performance. Some of the complications outcomes may have been more related to patient factors or to anaesthesia. The variables registered in GallRiks are defined to focus on patient-related outcome, not the surgical success. This may have contaminated the results.

Furthermore, the inclusion of intraoperative ERCP for patients presenting with common bile duct stones contributed to a longer total operation time. Nevertheless, due to the substantial volume of procedures, this factor is not anticipated to materially impact the overall findings. Additionally, the focus on operation time as a surrogate for the learning curve might be a rough estimate of the needed surgical volume. Hence, patient outcomes in terms of complications should be considered with other outcomes like readmission rates and quality of life, providing a better overall representation of surgical success. However, in this study complication rate is considered the most clinically relevant outcome measure when determining the readiness for independent practice, but this is influenced by numerous factors other than the experience of the surgeon. Nevertheless, by defining a reduction of the complication rate to a level equal to that of procedures completed by surgeons unaccompanied by an assistant, 250 procedures are needed to acquire sufficient safety to give a general recommendation of the number of procedures needed to pass the learning curve.

Future studies should explore whether simulation training can shorten the learning curve, particularly for operative efficiency in cholecystectomies, since simulation training is not mandatory in the Swedish trainee curriculum. It is suggested that it be included in the trainee curriculum as something trainees need to practise regularly throughout their postgraduate education. It is already well established that practising a skill in simulations makes for more efficient performance [21–27]. Furthermore, investigating the impact of surgeon-specific factors like prior experience with related procedures and patient-specific factors, e.g., obesity or comorbidities, could refine the classification tree model. Comparative studies across countries with differing training structures and surgical volumes could clarify whether Sweden’s prolonged learning curve is unique.

## Supplementary Information

Below is the link to the electronic supplementary material.

Supplementary file1 (PDF 192 KB)—Online Appendix 1. Tree diagram presenting key predictors of operation time by splitting the data into subgroups, with each node representing significant factor. First split was surgical approach (laparoscopic cholecystectomy was faster with a mean operation time of 93 min). The second split showed that acute cholecystitis cases took longer time compared to elective cases. The third split showed that mean operation time decreased with increased surgical volume

Supplementary file2 (PNG 202 KB)—Online Appendix 2. Tree diagram presenting key predictors of surgical complications by splitting the data into subgroups, with each node representing significant factor

Supplementary file3 (JPG 56 KB)—Online Appendix 3. Flowchart of cohort

## References

[1] Keus F, Gooszen HG, van Laarhoven C (2010) Open, small-incision, or laparoscopic cholecystectomy for patients with symptomatic cholecystolithiasis. An overview of Cochrane Hepato-Biliary Group reviews. Cochrane Database Syst Rev. 10.1002/14651858.CD008318 PMC7180153 20091665

[2] Soper NJ, Stockmann PT, Dunnegan DL, Ashley SW (1992) Laparoscopic cholecystectomy the new ‘gold standard’? Arch Surg 127(8):917–923 10.1001/archsurg.1992.01420080051008 1386505

[3] Brunt LM, Deziel DJ, Telem DA, Strasberg SM, Aggarwal R, Asbun H et al (2020) Safe cholecystectomy multi-society practice guideline and state of the art consensus conference on prevention of bile duct injury during cholecystectomy. Ann Surg 272(1):3–23. 10.1097/SLA.0000000000003791 32404658

[4] Ericsson KA (2004) Deliberate practice and the acquisition and maintenance of expert performance in medicine and related domains. Acad Med 79(10):S70–S81 10.1097/00001888-200410001-00022 15383395

[5] Soomro NA, Hashimoto DA, Porteous AJ, Ridley CJA, Marsh WJ, Ditto R et al (2020) Systematic review of learning curves in robot-assisted surgery. BJS Open 4(1):27–44 10.1002/bjs5.50235 PMC6996634 32011823

[6] Mán E, Németh T, Géczi T, Simonka Z, Lázár G (2016) Learning curve after rapid introduction of laparoscopic appendectomy: are there any risks in surgical resident participation? World J Emerg Surg 11(1):17 10.1186/s13017-016-0074-5 PMC4855767 27148395

[7] Delgado-Miguel C, Miguel-Ferrero M, San Basilio M, Ramírez C, Camps J, Hernández OF (2023) How does the number of training years in pediatric surgery affect appendectomy outcomes? Pediatr Surg Int 39(1):168 10.1007/s00383-023-05453-7 37029305

[8] Kim SY, Hong SG, Roh HR, Park SB, Kim YH, Chae GB (2010) Learning curve for a laparoscopic appendectomy by a surgical trainee. J Korean Soc Coloproctology 26(5):324 10.3393/jksc.2010.26.5.324 PMC2998026 21152134

[9] Svensk Kirurgisk Förening (2022) Utbildningsbok för specialiseringstjänstgöring i kirurgi. Svensk Kirurgisk Förening, Stockholm

[10] Williams RG, Swanson DB, Fryer JP, Meyerson SL, Bohnen JD, Dunnington GL et al (2019) How many observations are needed to assess a surgical trainee’s state of operative competency? Ann Surg 269(2):377–382 10.1097/SLA.0000000000002554 29064891

[11] Accreditation Council for Graduate Medical Education (ACGME) (2023) Program requirements for graduate medical education in general surgery. Chicago: ACGME. Available from: https://www.acgme.org/globalassets/pfassets/programrequirements/440_generalsurgery_2023.pdf

[12] Voitk AJ, Tsao SGS, Ignatius S (2001) The tail of the learning curve for laparoscopic cholecystectomy. Am J Surg 182(3):250–253 10.1016/s0002-9610(01)00699-7 11587686

[13] Waage A, Nilsson M (2006) Iatrogenic bile duct injury: a population-based study of 152 776 cholecystectomies in the Swedish inpatient registry. Arch Surg 141(12):1207–1213 10.1001/archsurg.141.12.1207 17178963

[14] de’Angelis N, Catena F, Memeo R, Coccolini F, Martínez-Pérez A, Romeo OM et al (2021) 2020 WSES guidelines for the detection and management of bile duct injury during cholecystectomy. World J Emerg Surg 16(1):30 10.1186/s13017-021-00369-w PMC8190978 34112197

[15] Tornqvist B, Stromberg C, Persson G, Nilsson M (2012) Effect of intended intraoperative cholangiography and early detection of bile duct injury on survival after cholecystectomy: population based cohort study. BMJ 345:e6457–e6457 10.1136/bmj.e6457 PMC3469410 23060654

[16] Nuzzo G, Giuliante F, Giovannini I, Ardito F, D’Acapito F, Vellone M et al (2005) Bile duct injury during laparoscopic cholecystectomy: results of an Italian national survey on 56 591 cholecystectomies. Arch Surg 140(10):986–992 10.1001/archsurg.140.10.986 16230550

[17] Enochsson L, Thulin A, Österberg J, Sandblom G, Persson G (2013) The Swedish registry of gallstone surgery and endoscopic retrograde cholangiopancreatography (GallRiks): a nationwide registry for quality assurance of gallstone surgery. JAMA Surg 148(5):471–478 10.1001/jamasurg.2013.1221 23325144

[18] Kass GV (1980) An exploratory technique for investigating large quantities of categorical data. J R Stat Soc Ser C Appl Stat 29(2):119–127

[19] Molloy M, Bower RH, Hasselgren PO, Dalton BJ (1999) Cholangiography during laparoscopic cholecystectomy—cumulative sum analysis of an institutional learning curve. J Gastrointest Surg 3(2):185–188 10.1016/s1091-255x(99)80031-6 10457344

[20] Giger UF, Michel JM, Opitz I, Inderbitzin DT, Kocher T, Krähenbühl L (2006) Risk factors for perioperative complications in patients undergoing laparoscopic cholecystectomy: analysis of 22,953 consecutive cases from the Swiss Association of Laparoscopic and Thoracoscopic Surgery Database. J Am Coll Surg 203(5):723–728 10.1016/j.jamcollsurg.2006.07.018 17084335

[21] Debru E, Dawson A, Leibman S, Richardson M, Glen L, Hollinshead J et al (2005) Does routine intraoperative cholangiography prevent bile duct transection? Surg Endosc 19(4):589–593 10.1007/s00464-004-8711-6 15759189

[22] Aggarwal R, Ward J, Balasundaram I, Sains P, Athanasiou T, Darzi A (2007) Proving the effectiveness of virtual reality simulation for training in laparoscopic surgery. Ann Surg 246(5):771–779 10.1097/SLA.0b013e3180f61b09 17968168

[23] Malik AA, Khan MS, Gondal UR, Ali Rahman U, Yousef A, Jabran M (2024) Learning curve of laparoscopic cholecystectomy: a risk-adjusted cumulative sum (CUSUM) analysis of 780 cases operated by surgical residents. Surg Endosc 38(2):734–740

[24] Madayag RM, Meier AH, Kuo MC, Laidlaw GL, Herrera N, Bader SG (2024) Learning curve for robotic-assisted cholecystectomy. JAMA Surg 159(7):821–822 10.1001/jamasurg.2024.1221 PMC11112490 38776095

[25] Kim J, Kim C, Kim I, Kim H, Kim J, Kim K (2024) Comparative analysis of learning curves in robotic versus laparoscopic cholecystectomy. Cureus 16(8):e67504 10.7759/cureus.67468 PMC11339721 39176181

[26] Ahlberg G, Enochsson L, Gallagher AG, Hedman L, Hogman C, McClusky DA 3rd et al (2007) Proficiency-based virtual reality training significantly reduces the error rate for residents during their first 10 laparoscopic cholecystectomies. Am J Surg 193(6):797–804 10.1016/j.amjsurg.2006.06.050 17512301

[27] Seymour NE, Gallagher AG, Roman SA, O’Brien MK, Bansal VK, Andersen DK et al (2002) Virtual reality training improves operating room performance: results of a randomized, double-blinded study. Ann Surg 236(4):458–463 (**discussion 463-464**) 10.1097/00000658-200210000-00008 PMC1422600 12368674

