# Supplementary material for: Learning curve and patient outcomes in gallstone surgery: a national register-based cohort study
Source: Surg Endosc. 2026 Jul 20;40(10):8561–7. doi: 10.1007/s00464-026-13073-0 (PMC13633137; doi:10.1007/s00464-026-13073-0)
Supplement: Supplementary file 1 [file 464_2026_13073_MOESM1_ESM.pdf]

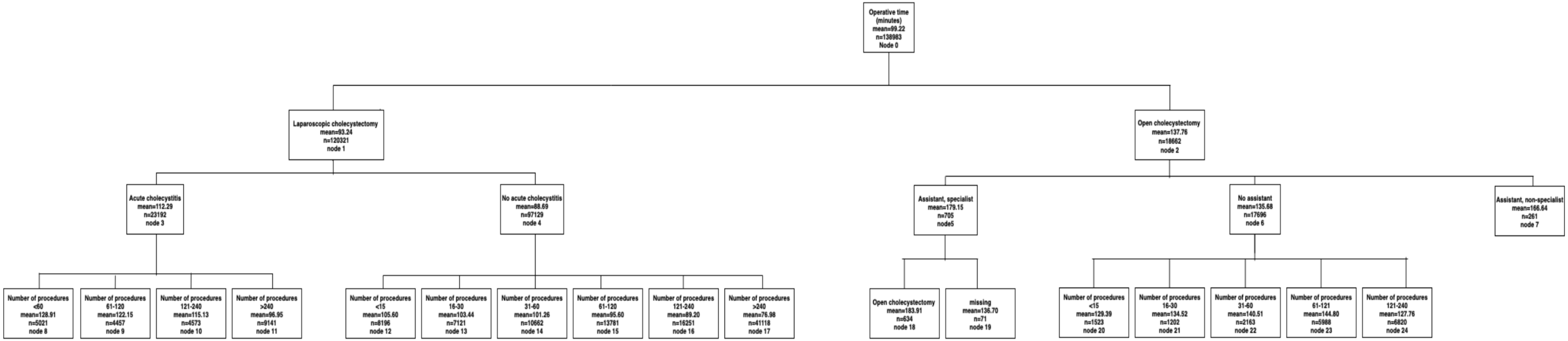

Operative time (minutes) mean=99.22 n=138983 Node 0
Laparoscopic cholecystectomy mean=93.24 n=120321 node 1
Open cholecystectomy mean=137.76 n=18662 node 2
Acute cholecystitis mean=112.29 n=23192 node 3
No acute cholecystitis mean=88.69 n=97129 node 4
Assistant, specialist mean=179.15 n=705 node5
No assistant mean=135.68 n=17696 node 6
Assistant, non-specialist mean=166.64 n=261 node 7
Number of procedures <60 mean=128.91 n=5021 node 8
Number of procedures 61-120 mean=122.15 n=4457 node 9
Number of procedures 121-240 mean=115.13 n=4573 node 10
Number of procedures >240 mean=96.95 n=9141 node 11
Number of procedures <15 mean=105.60 n=8196 node 12
Number of procedures 16-30 mean=103.44 n=7121 node 13
Number of procedures 31-60 mean=101.26 n=10662 node 14
Number of procedures 61-120 mean=95.60 n=13781 node 15
Number of procedures 121-240 mean=89.20 n=16251 node 16
Number of procedures >240 mean=76.98 n=41118 node 17
Open cholecystectomy mean=183.91 n=634 node 18
missing mean=136.70 n=71 node 19
Number of procedures <15 mean=129.39 n=1523 node 20
Number of procedures 16-30 mean=134.52 n=1202 node 21
Number of procedures 31-60 mean=140.51 n=2163 node 22
Number of procedures 61-121 mean=144.80 n=5988 node 23
Number of procedures 121-240 mean=127.76 n=6820 node 24
