# Supplementary material for: Learning curve and patient outcomes in gallstone surgery: a national register-based cohort study
Source: Surg Endosc. 2026 Jul 20;40(10):8561–7. doi: 10.1007/s00464-026-13073-0 (PMC13633137; doi:10.1007/s00464-026-13073-0)
Supplement: Supplementary file 2 [file 464_2026_13073_MOESM3_ESM.pdf]

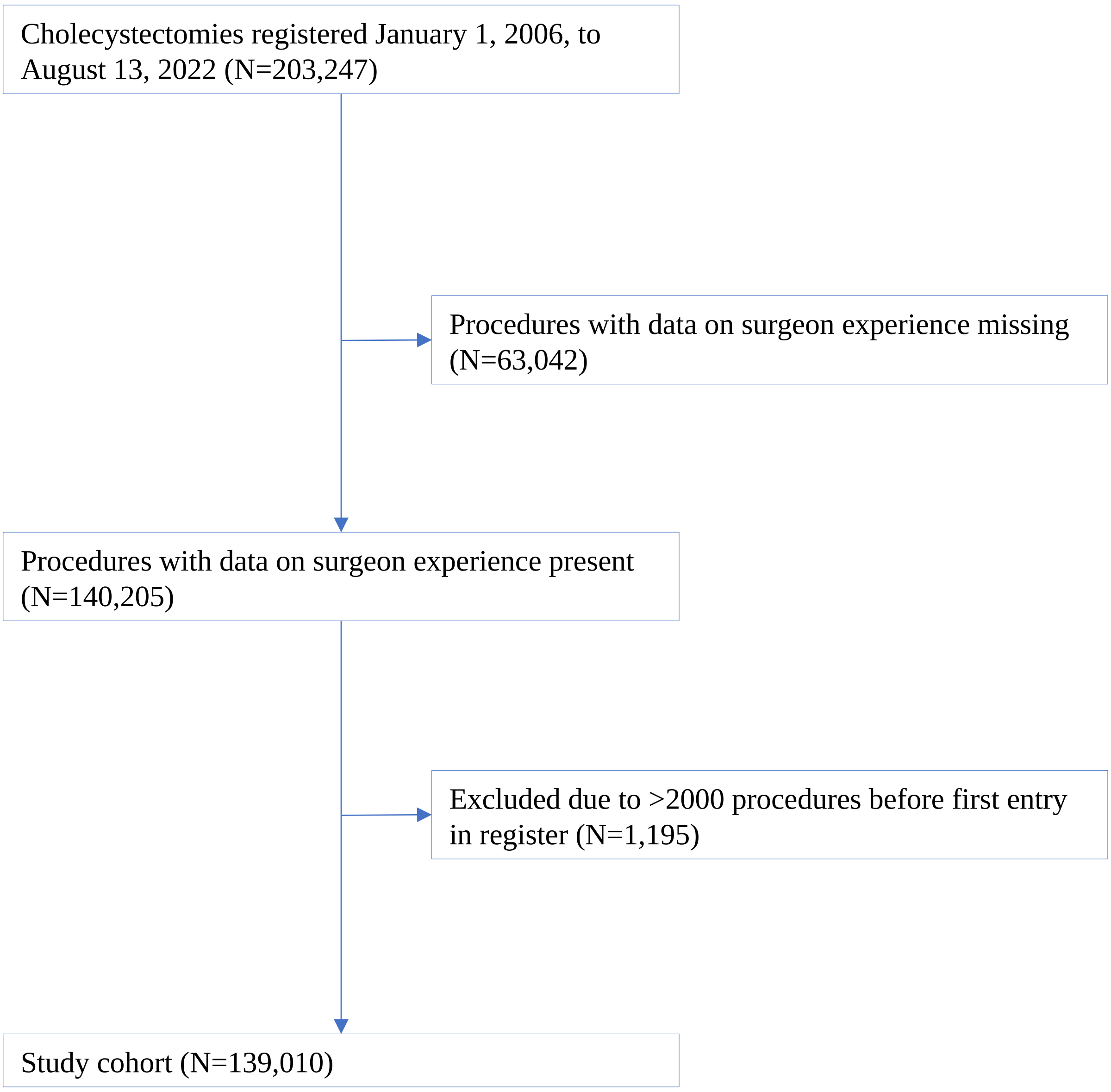
Cholecystectomies registered January 1, 2006, to August 13, 2022 (N=203,247)
Procedures with data on surgeon experience missing (N=63,042)
Procedures with data on surgeon experience present (N=140,205)
Excluded due to >2000 procedures before first entry in register (N=1,195)
Study cohort (N=139,010)
